# Development of a human antibody fragment directed against the alpha folate receptor as a promising molecule for targeted application

**DOI:** 10.1080/10717544.2021.1943055

**Published:** 2021-07-08

**Authors:** Nattihda Parakasikron, Chatchai Chaotham, Pithi Chanvorachote, Chanida Vinayanuwattikun, Visarut Buranasudja, Pornchanok Taweecheep, Kannika Khantasup

**Affiliations:** a The Medical Microbiology Program, Graduate School, Chulalongkorn University, Bangkok, Thailand; b Department of Biochemistry and Microbiology, Faculty of Pharmaceutical Sciences, Chulalongkorn University, Bangkok, Thailand; c Cell-Based Drug and Health Product Development Research Unit, Faculty of Pharmaceutical Sciences, Chulalongkorn University, Bangkok, Thailand; d Department of Pharmacology and Physiology, Faculty of Pharmaceutical Sciences, Chulalongkorn University, Bangkok, Thailand; e Vaccines and Therapeutic Proteins Research Group, the Special Task Force for Activating Research (STAR), Faculty of Pharmaceutical Sciences, Chulalongkorn University, Bangkok, Thailand; f Division of Medical Oncology, Department of Medicine, Faculty of Medicine, Chulalongkorn University, Bangkok, Thailand

**Keywords:** Non-small-cell lung cancer (NSCLC), targeted therapy, alpha folate receptor (FRα), variable domain of a heavy-chain (VH), phage display

## Abstract

Alpha folate receptor (FRα) is currently under investigation as a target for the treatment of patients with non-small-cell lung cancer (NSCLC), since it is highly expressed in tumor cells but is largely absent in normal tissue. In this study, a novel human variable domain of a heavy-chain (VH) antibody fragment specific to FRα was enriched and selected by phage bio-planning. The positive phage clone (3A102 VH) specifically bound to FRα and also cross-reacted with FRβ, as tested by ELISA. Clone 3A102 VH was then successfully expressed as a soluble protein in an *E. coli* shuffle strain. The obtained soluble 3A102 VH demonstrated a high affinity for FRα with affinity constants (K_aff_) values around 7.77 ± 0.25 × 10^7^ M^−1^, with specific binding against both FRα expressing NSCLC cells and NSCLC patient-derived primary cancer cells, as tested by cell ELISA. In addition, soluble 3A102 VH showed the potential desired property of a targeting molecule by being internalized into FRα-expressing cells, as observed by confocal microscopy. This study inspires the use of phage display to develop human VH antibody (Ab) fragments that might be well suited for drug targeted therapy of NSCLC and other FRα-positive cancer cells.

## Introduction

Lung cancer is the second most common cause of death from cancer in the world (Siegel et al., 2017; Woodman et al., 2021). It can be divided into the two types of non-small cell lung cancer (NSCLC) and small cell lung cancer (SCLC), which account for 85% and 15% of lung cancers, respectively (Shi et al., 2013). Overall, NSCLC has received attention in many studies because it is found in the majority of lung cancers that cause death and metastasize to other organs, such as the brain and liver (Tamura et al., 2015). Currently, targeted therapy is viewed as a potentially good approach for the treatment of NSCLC and other cancers (Sokolowska-Wedzina et al., 2017). However, this therapy requires the discovery of drug-targeting molecules specific to tumor-associated antigens (TAAs) or tumor antigens (TAs) in order to be more specific to cancer cells (Jäger et al., 2001; Ahmad et al., 2012; Iqbal & Iqbal, 2014)

In the case of NSCLC, TAAs that are interesting for use in clinical areas include alpha folate receptors (FRα), mucin 1, and the transforming growth factor-beta receptor (Kalli et al., 2008; Furler et al., 2018; Syrkina et al., 2019). Focusing on FRα, it is a membrane glycoprotein that is overexpressed on the surface of various tumor types, including NSCLC as well as pancreatic, ovarian, and breast cancers (Hartmann et al., 2007; Iwakiri et al., 2008; Kalli et al., 2008), whereas it is expressed at a low level on the apical surface of normal epithelial cells of the lungs, kidneys, choroid plexus, and uterus (Fernández et al., 2018). This restricted distribution makes FRα out of direct contact with the bloodstream, and so it is unable to be accessed with drug-conjugated folate-targeting agents, resulting in normal cells being less susceptible to cytotoxic drugs (Srinivasarao et al., 2015; Patel et al., 2016). In contrast, epithelial cancer cells overexpress FRα on their basal surface, where it is exposed to drug-conjugated folate-targeting agents in the bloodstream, leading to a more specific destruction of cancer cells (Toffoli et al., 1998; Allard et al., 2007; Brown Jones et al., 2008). For this reason, FRα has been viewed as a potential marker for both folic acid and FRα specific antibodies (Abs) to develop a diagnostic and targeted drug delivery system for discriminating between normal cells and FRα-overexpressing cancer cells (Ab et al., 2015; Sato & Itamochi, 2016).

According to Ab-drug conjugate (ADC)-based targeted therapeutic strategies, FRα-targeting monoclonal Antibody (mAb) conjugated with a cytotoxic drug could offer potential benefits, such as reducing the required therapeutic dose and avoiding the nonspecific cytotoxicity toward normal tissues. For example, Mirvetuximab soravtansine, a humanized FRα-targeting Ab conjugated with the maytansinoid DM4 drug, can induce cell-cycle arrest and cell death by targeting the microtubules of cancer cells (Kovtun et al., 2006; Lambert, 2013; Moore et al., 2018). This FRα-specific mAb is currently in clinical use for the treatment of NSCLC and ovarian cancers (Konner et al., 2010; Shi et al., 2013; Ponte et al., 2016).

Recently, phage display has emerged as a new technique that displays an Ab fragment on the surface of the bacteriophage (Bazan et al., 2012). This technique allows bio-panning to screen for Abs specific to the target of interest, and does not require any laboratory animals (Azzazy & Highsmith, 2002). Moreover, the phages can express just the variable domain of a heavy-chain (VH) Ab, and these have been discovered from camelids, nurse sharks, and human VH synthetics (Davies & Riechmann, 1996; Hairul Bahara et al., 2016). Due to its smaller size, the VH has many advantages over an intact Ab, such as a low immunogenicity, good penetrance into solid tumors, low tumor to background ratio, and the ability to access cryptic epitopes (Harmsen & De Haard, 2007). In addition, the VH has a high serum stability and can resist a wide range of pH and temperatures compared with peptides, which are another popular type of targeting molecule (Thundimadathil, 2012; Bates & David, 2019 Apr 9). These benefits open up a new idea for cancer treatment by conjugating VH Ab molecules with toxic-drugs or radioisotopes, to result in not only a high tumor penetration but also a faster blood clearance, which would reduce the undesired nonspecific drug cytotoxic effects (Harmsen & De Haard, 2007; Rodriguez-Fernandez et al., 2019). In this study, a phage display library was used to select a human VH Ab directed against FRα that is overexpressed on NSCLC. The VH specific to FRα was evaluated for its binding activity and cell-internalization after binding. The obtained VH could be used for further development of diagnosis or targeted drug therapy that is specific to NSCLC and other FRα-positive cancer cells.

## Materials and methods

### Materials and bacterial strain

The recombinant human alpha and beta folate receptor proteins (rhFRα and rhFRβ) were purchased from Sino Biological Inc. (Eschborn, Germany). The human domain antibody library (Dab) (Source Bioscience, Nottingham, UK) was used for selecting the VH phage specific to rhFRα. The Abs used in this study included: rabbit anti-human FRα polyclonal ab (Sino Biological, Germany), anti-M13 Ab-HRP conjugate (Sino Biological, Beijing), protein A-HRP conjugate (Abcam, U.K.), mouse anti-His-tag (Cell Signaling, USA), mouse anti-His-tag alkaline phosphatase (AP) conjugate (Cell Signaling, USA), goat anti-mouse IgG-FITC conjugate (Merck, Germany), and protein A-FITC conjugate (Abcam, U.K.). *Streptococcus suis* serotype 2 specific VH, hereafter called the irrelevant soluble VH, was produced in-house. The *E. coli* Shuffle® T7 competent cell strain (New England Biolabs, USA) was used to express soluble VH. This strain was cultured in Terrific Broth (TB) supplemented with 50 µg/mL of kanamycin at 30 °C.

### Cell lines and culture conditions

The human NSCLC H292, A549 cell lines, human breast cancer MDA-MB-231 (FRα-expressing cells) and the human skin fibroblast BJ cells (FRα non-expressing cells) were obtained from the American Type Culture Collection (Manassas, VA, USA).The ethically approved patient-derived primary NSCLC cells ECL-08, -10, -12, -16, -17, and -20 (IRB 365/62) were kindly cultured and provided by Prof. Dr. Pithi Chanvorachote (Faculty of Pharmaceutical Sciences, Chulalongkorn University, Thailand). The A549, MDA-MB-231 and BJ cells were cultured in DMEM, while H292 cells were cultured in RPMI, each supplemented with 10% (v/v) heat-inactivated fetal bovine serum, 1% L-glutamine, 1% penicillin, and 1% streptomycin (Gibco, Gaithersburg, MA, USA). Cells were maintained under 5% CO_2_ at 37 °C until at 70-80% confluence before using for experiments.

All cell lines used in experiments were confirmed for the expression of FRα by cell-based ELISA and cell immunofluorescence using a rabbit anti-hFRα polyclonal antibody.

### Bio-panning

The Human Dab library was screened for VH Abs specific to rhFRα. The phage library was amplified to 2.1 × 10^11^ plaque forming units (pfu) and subjected to seven rounds of bio-panning. Firstly, the phage library was pre-absorbed with 1% (w/v) bovine serum albumen (BSA) at room temperature (RT) for 60 min to remove phages with specific binding to the BSA-based blocking buffer used in the panning system. Six wells of 96-well plates were coated with rhFRα at 2.5 µg/well and incubated overnight at 4 °C. The wells were washed with phosphate buffered saline pH 7.4 (PBS) five times and then blocked with 3% (w/v) BSA at 37 °C for 1 h. After removing the blocking solution, 2.3 × 10^10^ pfu of pre-adsorbed phage were added in each well. The wells were further incubated on a platform shaker with gentle shaking for 30 min at RT and then stood still for 60 min at RT. The wells were then washed with PBS containing 0.1% (v/v) Tween-20 (PBST) 15 times, followed by washing twice with PBS. The elution of phage binding was performed by adding 50 µL of trypsin (0.5 mg/mL) and left on a platform shaker with gentle shaking for 1 h at RT. The eluted phages (output) were determined for titration and amplified to be input phage for the next round by infecting *E. coli* TG1 as previously described (Lee et al., 2007). The pre-absorption step was applied as the first step in each round. Stringency of selection was performed by increasing the washing time and % (v/v) Tween-20 in the washing buffer, as summarized in Table 1.

### Polyclonal phage ELISA

Amplified phages (input) from each round of bio-panning were screened for binding activity to rhFRα using a polyclonal phage ELISA. For this, a 96-well plate was coated overnight with 2.5 µg/well of rhFRα or rhFRβ or BSA at 4 °C. The wells were washed with PBS and blocked with 2% (w/v) skim milk in 0.05% PBST (2%MPBST) at 37 °C for 1 h. After removing the blocking solution, the amplified phages of each round were diluted in 2%MPBST, added into each well, and incubated at 37 °C for 1 h. The phage suspensions were discarded and the wells were washed five times with 0.05% PBST. To detect bound phage, a 1:2,000-fold dilution of anti-M13 Ab-HRP conjugate in 2%MPBST was added to the wells and incubated at 37 °C for 1 h. After washing, bound-phages were detected using the BioFX^®^ TMB substrate (Surmodics IVD, Inc., Eden Prairie, USA) at RT for 30 min in the dark, with the reaction being stopped by the addition of BioFX^®^ 450 nm liquid Nova-stop solution (Surmodics IVD, Inc., Eden Prairie, USA). The absorbance of each well was read at 450 nm (A_450_) using a CLARIOstar^®^ microplate reader (BMG LABTECH, Singapore). The uncoated wells served as a negative control.

### Monoclonal phage ELISA

The eluted phages from the seventh bio-panning round were transfected to *E. coli* TG1. A total of 145 individual clones were picked for screening using a monoclonal phage ELISA. Briefly, a single colony was cultured in cell culture microplates at 37 °C with shaking at 200 rpm for 3 h. After incubation, 4 × 10^8^ pfu/well of helper phage was added and incubated for 1 h at 37 °C. The plates were then centrifuged at 2000 × g for 15 min and resuspended in 200 µL 2x tryptic soy broth supplemented with 100 µg/mL ampicillin and 50 µg/mL kanamycin and incubated at 25 °C with shaking at 200 rpm overnight. The plates were centrifuged to harvest the amplified phage in the supernatant. Each 96-well plate was coated with 0.8 µg/well of rhFRα protein. After the blocking and washing steps, the individual amplified phage supernatants (50 µL) were diluted with 50 µL of 5%MPBST. The positive phage clones were detected and performed in the same manner as described in polyclonal ELISA system. Phage clones that gave a three-fold greater signal (A_450_) in the wells coated with rhFRα than that in the uncoated wells were selected as positive phage clones.

### Cross-reactivity test of phage clone

Positive phages selected from the monoclonal ELISA were tested for cross-reactivity with rhFRα, rhFRβ, and 3% (w/v) BSA. Each 96-well plate was coated with 0.8 µg/well of rhFRα or rhFRβ or 3% BSA. After blocking with 2%MPBST, 50 µL of positive phage diluted in 50 µL of 5%MPBST was added in each well and incubated at 37 °C for 1 h. The binding activity of the phages was then evaluated in the same manner as described in the polyclonal ELISA section, using uncoated wells as a negative control.

### Examination of the VH amino acid sequence

The phagemid from each positive clone was extracted to determine the DNA sequence of the VH in the recombinant phagemid. The VH sequencing was performed using the pR2-vector specific primers LMB3: 5′-CAGGAAACAGCTATGAC-3′. The DNA sequences and the deduced amino acid sequences were compared with the DNA sequences in the GenBank sequence database to determine the complementarity-determining regions (CDR) and framework.

### Expression and purification of the soluble VH

The 3A102 positive phage clone (3A102 VH) was used for expression as soluble 3A102 VH in an *E. coli* expression system. The 3A102 VH gene was synthesized by the Invitrogen company (Genscript, USA) and re-cloned into the pET-28b vector (Genscript, USA) to construct a 6xHis-tag fused 3A102 VH recombinant gene. After that, the 3A102 recombinant plasmid was transformed into *E. coli* Shuffle and then cultured in TB supplemented with 50 µg/mL of kanamycin and incubated with shaking at 30 °C until the OD at 600 nm (OD_600_) reached ∼0.7-0.8. Then, the antibody expression was induced by 0.5 mM of isopropyl-1-thio-β-D-galactopyranoside and incubated at 30 °C for 21 h. After incubation, the culture was collected and centrifuged at 5,000 rpm 4 °C for 10 min. The pellet was resuspended in lysis buffer [150 mM NaCl, 1% (v/v) Triton x-100, 50 mM Tris-HCl, and 20 mM imidazole, pH 7.4]. Cytoplasmic soluble protein was extracted by sonication (10 s pulse cycles for 3 min with 35% amplitude) on ice. The cell lysates were centrifuged at 5,000 rpm, 4 °C for 10 min to remove insoluble fractions. The soluble 3A102 VH in lysis buffer was enriched by immobilized metal affinity chromatography. Briefly, the soluble proteins in lysis buffer were added to a nickel-nitrilotriacetic acid agarose column (GE, USA) at a 0.5 mL/min flow velocity, and then the column was washed with washing buffer (40 mM imidazole, 0.5 M NaCl, and 20 mM sodium phosphate, pH 7.4). The soluble 3A102 VH bound to the column was then eluted with 400 mM imidazole in a washing buffer. The purity of the soluble 3A102 VH was evaluated by 15% (w/v) sodium dodecyl sulphate-polyacrylamide gel electrophoresis (SDS-PAGE) under a reducing condition and, for western blots, was detected by mouse anti-His-tag AP conjugate and BCIP/NBT AP substrate (Surmodics IVD, Inc., Eden Prairie, USA).

### Bioactivity determination of soluble VH

The binding ability of soluble 3A102 VH with FRα was tested by ELISA. Briefly, 1 µg/well of rhFRα was coated overnight at 4 °C. The wells were blocked with 5%MPBST and then two-fold concentrations of 3A102 VH from 1.75–28 µg/mL were added to the wells and incubated for 1 h at 37 °C. After washing, the binding activity of soluble 3A102 VH to the rhFRα was detected using the protein A-HRP conjugate (1:1500 in 2%MPBST), with the bound VH being detected using the TMB substrate in the same manner as described in the polyclonal ELISA section.

### Affinity test

The affinity constant (K_aff_) was determined by indirect ELISA as previously reported (Beatty et al., 1987). In brief, 96-well plates were coated with two-fold concentrations from 0.5–2 µg/well of rhFRα antigen. After overnight incubation, the plate was blocked with 2%MPBST and washed. The wells were then incubated with 50 µL of different concentrations (2.5–40 µg/mL) of soluble 3A102 VH for 1 h at 37 °C, washed with PBST, and then incubated with 1:1500 protein A-HRP conjugate in 2%MPBST. The bound VH was detected using the TMB substrate in the same manner as described in polyclonal ELISA section. The affinity constant (K_aff_)was calculated from K_aff_ = (*n* − 1)/2(n[Ab’]t − [Ab]t)

### Cell-based ELISA

The FRα-expressing cell lines, MDA-MB 231, H292, A549 and NSCLC patient-derived primary cancer cells were used for FRα-binding test while the BJ fibroblast cell line that does not express FRα was used as a negative control. The binding ability of the soluble 3A102 VH against FRα expressed on the cell surface was evaluated using a cell-based ELISA as described below. Briefly, 8,000 cells/well of positive or negative FRα expressing cells were seeded in a 96-well cell culture plate and allowed to attach to the well surface. After growing in media for 24 h, cells were fixed with 4% formaldehyde and blocked with 5% (w/v) skim milk in PBS (MPBS). After that, the soluble 3A102 VH (2.5–40 µg/mL) was added to each well and incubated at 37 °C for 1 h before being washed with PBS three times. The binding ability of the VH Ab to FRα was detected using a 1:1000 dilution of the protein A-HRP conjugate. After washing, the bound VH was detected using the TMB substrate in the same manner as described in the polyclonal ELISA section.

### Immunofluorescence assay (IFA)

The targeting ability of soluble 3A102 VH against FRα expressed on the cell surface was also evaluated using an IFA. Here, 8,000 cells/well of MDA-MB-231 and H292 cells were seeded in eight-well chamber slides. After seeding and fixation, as described in the cell-based ELISA section, the cells were incubated with 50 µg/mL of soluble 3A102 VH or the irrelevant soluble VH at 37 °C for 1 h. The BJ cells incubated with soluble 3A102 VH were used as a negative control. The VH Abs bound on the cell surface were detected using 1:200 of mouse anti-His-tag and then incubated with 1:200 of goat anti-mouse IgG-FITC conjugate. After washing to remove nonspecific bound Abs, the nuclei were stained with Hoechst 33342 and imaged with scanning laser confocal microscopy (SLCM;Olympus Fluoview FV10i, Olympus Corporation, Japan).

### Cell internalization assay

The FRα expressing H292 cell line was used to evaluate the internalization of soluble VH. In brief, 10,000 cells/well were seeded on eight-well chamber slides and incubated at 37 °C in 5% CO_2_ for 24 h. Then, 50 µg/mL of soluble 3A102 VH or the irrelevant soluble VH were incubated with the H292 cells for 3 h at 4 °C or 37 °C in 5% CO_2_ to allow cell internalization. The BJ cells incubated with soluble 3A102 VH were used as the negative control. After washing, cells were fixed with 4% paraformaldehyde and permeabilized with 0.5% (v/v) Triton X-100 for 5 min at RT, and then blocked with 3% (w/v) BSA at 37 °C for 1 h. To visualize antibody internalization, the cells were stained with 1:200 dilution of the protein A-FITC conjugate in 1% (w/v) BSA and nuclei were stained with Hoechst 33342 at 37 °C. The internalized fluorescent signals were imaged using SLCM.

### Statistical analysis

Data are expressed as the mean ± one standard deviation (SD). Comparisons between means were performed using an unpaired t test for independent samples. Statistical analysis was performed using the SPSS version 22.0 software (SPSS Inc., Chicago, IL, USA). Statistical significance was accepted at the *p* < 0.05 level.

## Results

### Enrichment of phage specific to rhFRα protein

**Figure 1.**
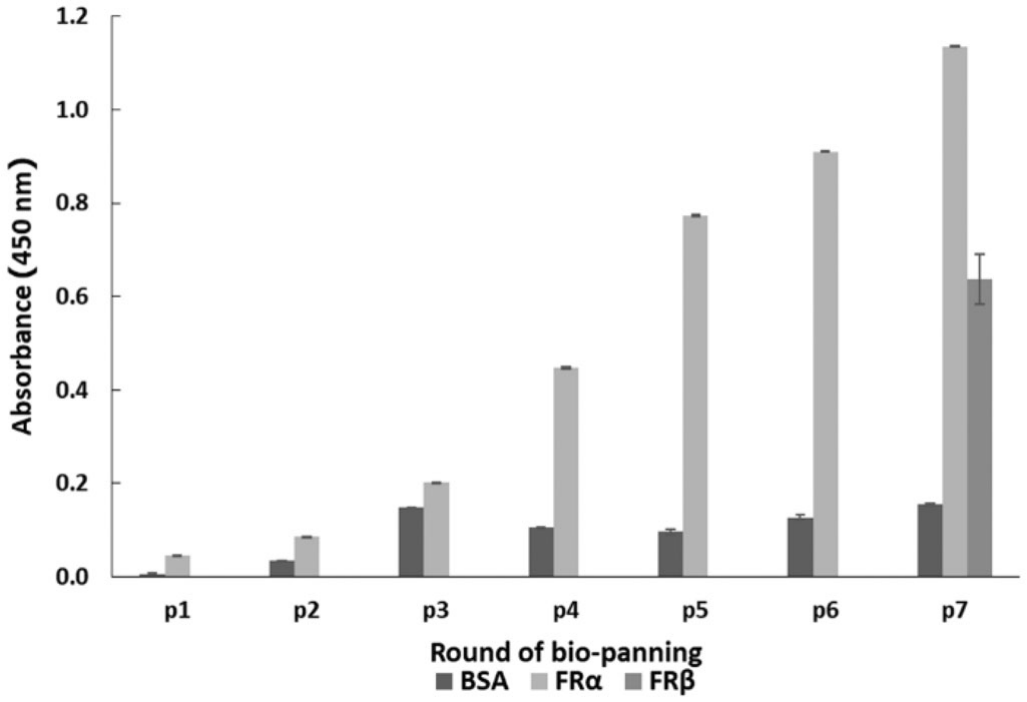
Polyclonal phage ELISA result for phage-binding (A_450_) to three different targets during seven rounds of bio-panning. Data are shown as the mean ± SD (*n* = 3).

Seven rounds of phage bio-panning with increasing selection stringency (see Table 1) were performed to highly enrich for rhFRα-specific binding phage. After each round of phage bio-panning, titration experiments showed a gradual increase in the output/input ratio of the eluted phage after each round. The eluted phage titration increased from 3.9 × 10^5^ pfu in the first round to 2.4 × l0^7^ pfu in the last round, with an enrichment of about 62-fold (Table 2). This result suggested a successful enrichment of specific phages against rhFRα. Meanwhile, the enriched phages of each round were tested for their ability to bind to rhFRα using a polyclonal phage ELISA. The ELISA result (Figure 1) showed an increased A_450_ signal to 1.137 in the seventh round, indicating that rhFRα-specific phages were effectively enriched for in the bio-panning process. However, we found that the panning could also enrich for phages specific to rhFRβ, another type of surface folate receptor.

**Table 1.** Concentration of the target rhFRα, number of washes, and concentration of Tween-20 in the wash buffer for the seven rounds of bio-panning.

Round	1	2	3	4	5	6	7
rhFRα (µg/well)	2.5	1.25	0.625	0.313	0.156	0.156	0.156
% (v/v) Tween-20	0.1	0.2	0.3	0.4	0.5	0.6	0.6
Number of washes	15	15	15	15	15	20	20

**Table 2.** Phage enrichment evaluation during seven rounds of bio-panning, as determined by phage titration.

Round	Input (pfu)	Output (pfu)
1	2.3 × 10^10^	3.9 × 10^5^
2	1.6 × 10^13^	5.8 × 10^7^
3	1.3 × 10^13^	2.7 × 10^7^
4	7.7 × 10^11^	2.6 × 10^7^
5	2.4 × 10^12^	4.3 × 10^7^
6	1.7 × 10^12^	2.1 × 10^7^
7	3.6 × 10^12^	2.4 × 10^7^
Enrichment		62-fold

### *Selection of FRα-specific phages* by monoclonal *phage ELISA*

**Figure 2.**
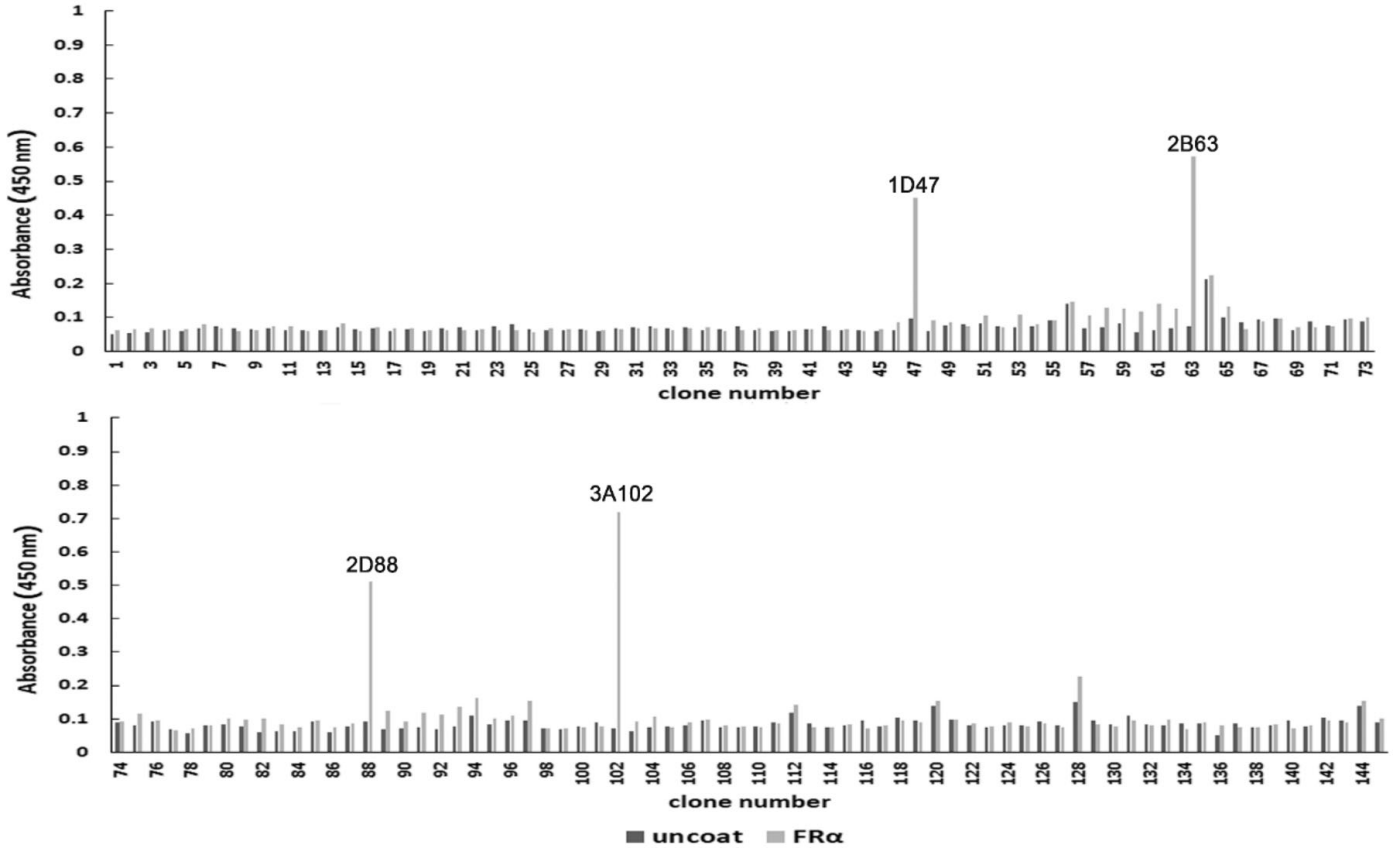
The rhFRα-specific phage binding analysis of 145 randomly selected clones from the seventh round of bio-panning.

After the seventh round of bio-panning, 145 phage clones were randomly selected from the eluted phages and their binding ability to rhFRα was analyzed using a monoclonal phage ELISA. Four clones (1D47, 2B63, 2D88, and 3A102) had the acceptable criterion of an A_450_ at least three-fold greater than that seen in the uncoated wells (Figure 2). Hence, these four phage clones were identified as positive and screened for cross-reactivity.

### Cross-reactivity of the four phage clones (1D47, 2B63, 2D88, and 3A102)

**Figure 3.**
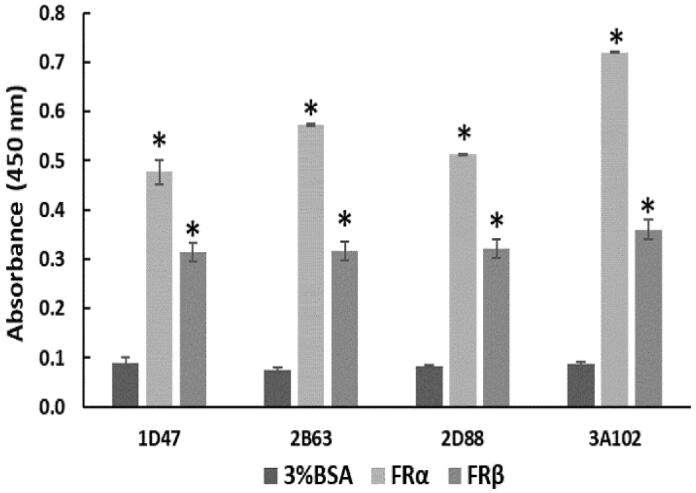
Cross-reactivity of the four positive phage clones against 3%BSA, rhFRα, and rhFRβ, as tested by ELISA. Data are shown as the mean ± SD (*n* = 3). *p < 0.05 compared to the negative control (uncoated well).

To determine the cross-reactivity of the four positive phage clones, they were tested against rhFRα, rhFRβ, and BSA. No cross reactivity to BSA, a blocking buffer used in the bio-panning, was observed. Among these four positive clones, 3A102 (3A102 VH) had the highest binding ability against rhFRα, but it and four clones also showed cross reactivity with rhFRβ (Figure 3).

### Examination of the amino acid sequence of the phage clones showing binding to FRα

The four selective positive clones (1D47, 2B63, 2D88, and 3A102) were examined to identify their VH sequences. The amino acid sequences of the four positive clones are shown in Table 3. Multiple sequence alignment revealed that clones 2B63 and 2D88 had an identical sequence, and all four clones had translational defects in the CDRs: namely amber stop codons (UAG).

**Table 3.** Amino acid sequence analysis of the four different phage clones of VH (1D47, 2B63, 2D88, and 3A102). Identical residues between the four positive clones are marked by (*). The amber stop codon is marked by (-).

	Framework-1	CDR1	Framework-2	CDR2
1D47	QVQLLESGGGLVQPGGSLRLSCAASG	F*LSH-Y*T	WVRQAPGKGLEWVS	T*GVHS
2B63		Y*FNS-A*G		S*SMAG
2D88		Y*FNS-A*G		S*NMRG
3A102		F*LSH-Y*T		T*GVHS
	Framework-3	CDR3	Framework-4	
1D47	GSTYYADSVKGRFTISRDNSKNTLYLQMNSLRAEDTAVYYCA	SYR*V**KS . . .. . HLKF	WGQGTLVTVSSAAA	
2B63		*VP*S**WAGLTAKPIRY		
2D88		*VP*S**WAGLTAKPIRY		
3A102		*KWFRE*FF . . LAPSLKS		

### Expression and purification of soluble VH

**Figure 4.**
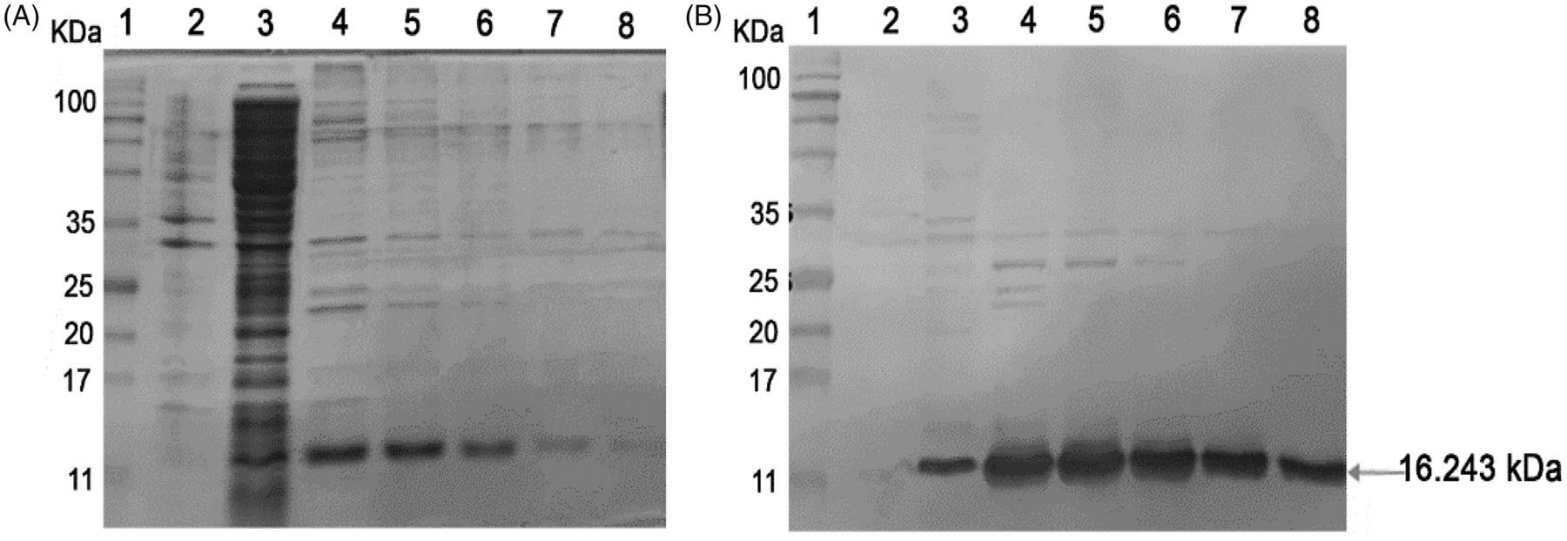
Soluble 3A102 VH expression and purification. (A) SDS-PAGE analysis of soluble 3A102 VH expressed in *E. coli* shuffle strains. Protein marker (MW, lane 1), total protein in pellet (lane 2), soluble fraction in cytoplasm (lane 3), and purified 3A102 VH in Eluted fractions 1–5 as lane 4–8, respectively. (B) Western blot analysis of the soluble 3A102 VH. Pre-stained protein marker (lane 1), total protein in pellet (lane 2), soluble fraction in the cytoplasm (lane 3), and purified 3A102 VH in Eluted fractions 1–5 as lane 4–8, respectively.

The 3A102 VH that showed the strongest binding activity (highest A_450_ signal) against the FRα in the monoclonal phage ELISA, was expressed as a soluble VH protein in the *E. coli* expression system. The sequencing data showed that 3A102 VH had an amber stop codon at CDR3, this amber codon was replaced with glutamine, before expressing as a soluble VH protein (soluble 3A102 VH) in the *E. coli* shuffle strain, so as to obtain disulfide bond formation and complete VH expression. The produced soluble 3A102 VH was then evaluated and confirmed for its expression and the purity after purification via SDS-PAGE and western blotting analyses, respectively. The soluble 3A102 VH was successfully expressed in a soluble form in the bacterial cytoplasm with a molecular weight of approximately 16.243 kDa (Figure 4(A,B) lane 3). The SDS-PAGE results also showed that the soluble 3A102 VH was purified with purity about 90%, after protein purification.

### Bioactivity determination of soluble VH

**Figure 5.**
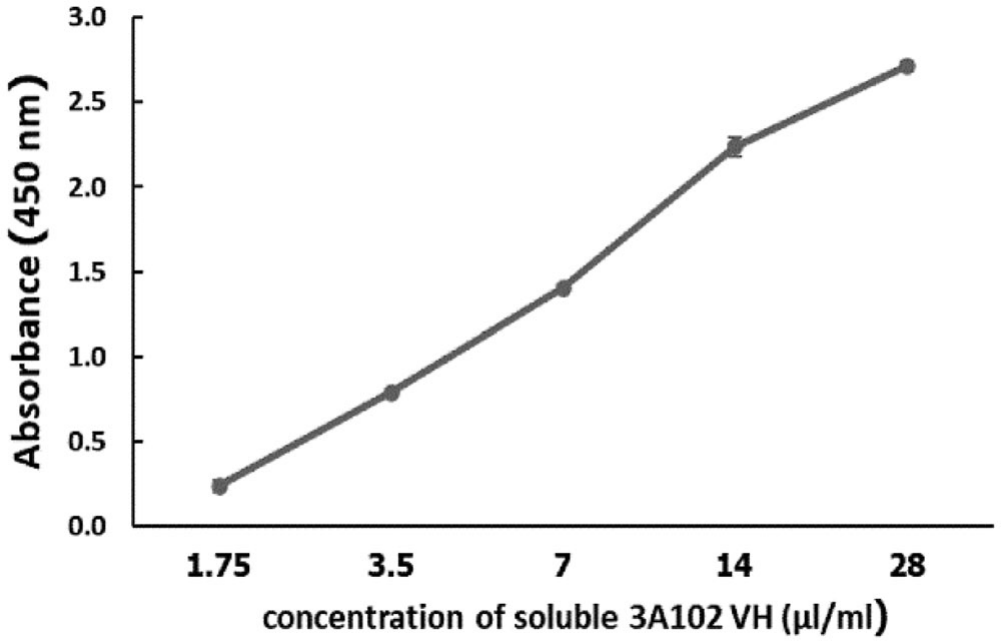
Binding assay of the purified soluble 3A102 VH against rhFRα, as evaluated by ELISA. Data are shown as the mean ± SD (*n* = 3).

To verify the binding ability of purified soluble 3A102 VH, the concentration from which the soluble 3A102 VH bound to rhFRα in the ELISA test was determined. As shown in Figure 5, Soluble 3A102 VH bound to the rhFRα antigen in a dose-dependent manner. Accordingly, soluble 3A102 VH still retains its binding activity against rhFRα after expression as a soluble protein in the *E. coli* Shuffle strain.

### Affinity of soluble VH

**Figure 6.**
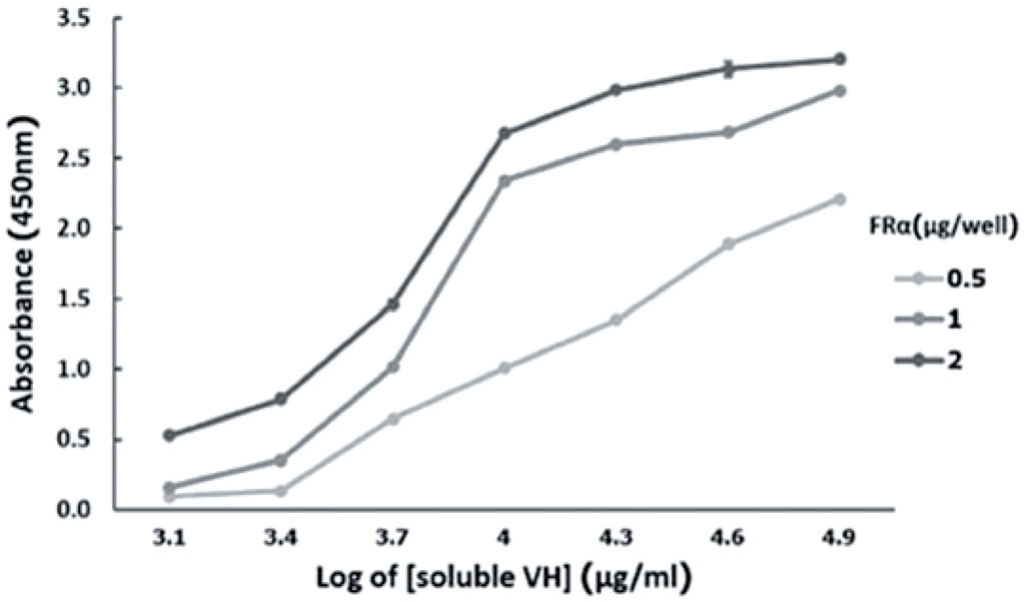
Affinity of soluble 3A102 VH antibody, as tested by ELISA, based on Beatty et al. (Beatty et al., 1987).

The affinity constant (K_aff_) is a parameter that shows the ability of the Ab to bind to its antigen. Determination of the K_aff_ from three different rhFRα concentrations (Figure 6), based on Beatty et al. (Beatty et al., 1987), revealed K_aff_ values for the soluble 3A102 VH to rhFRα to be around 7.77 ± 0.25 × 10^7^ M^−1^ (Table 4).

**Table 4.** Affinity constants of soluble 3A102 VH against recombinant FRα proteins, as determined by ELISA.

Ag (µg/well)	OD-50^a^	Ab at OD-50 (ng/mL)	K_aff_ (M^-1^)	Average K_aff_ (M-1) ± SD
0.5	0.68	89.04	7.8 × 10^7^	7.77 ± 0.25 × 10^7^
1	1.30	76.56	4.7 × 10^7^	
2	1.50	45.1	2.8 × 10^7^	

^a^OD-50 represents the half maximum optical density obtained for a given concentration of rhFRα ([Ag]) and the corresponding soluble 3A102 VH ([Ab]). The affinity constant (K_aff_) for each selected concentration of Ag and Ab was determined using the formula described in the Methods. Data are shown as the mean ± SD (*n* = 3).

### Evaluation of the binding ability of soluble VH to FRα on NSCLC cells

**Figure 7.**
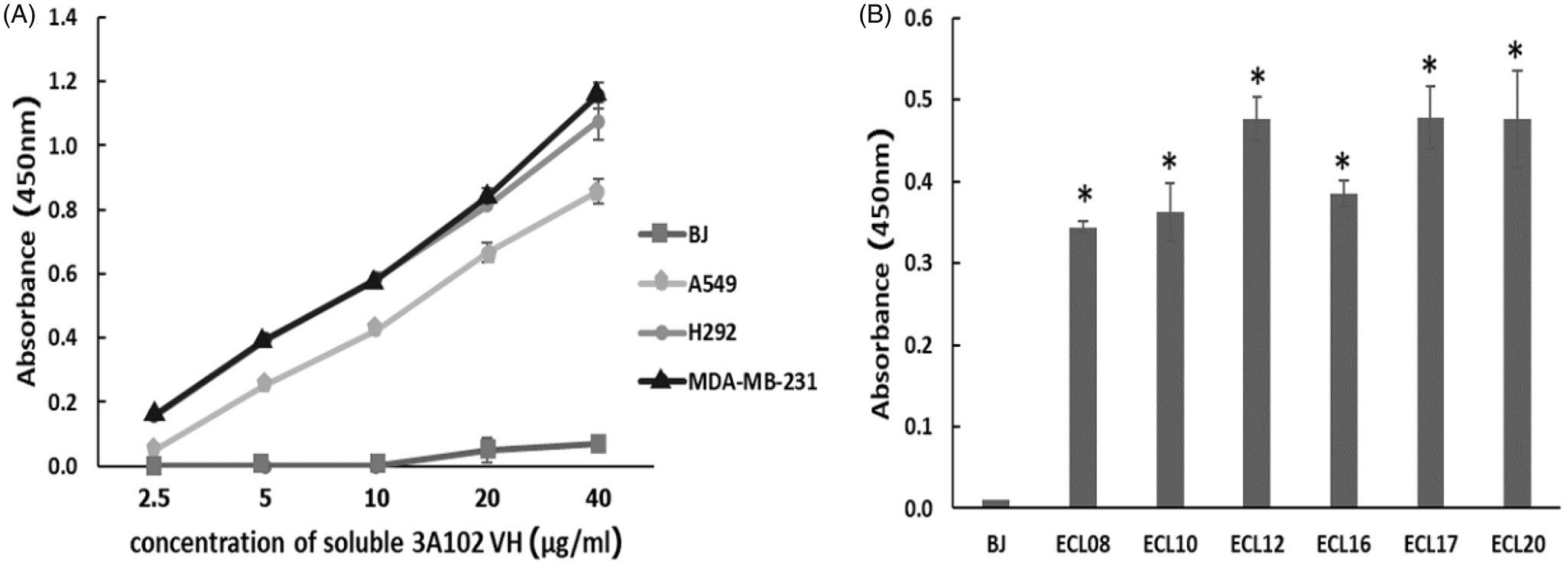
The binding activity of soluble 3A102 VH against FRα on cells surface, as evaluated by a cell-based ELISA. Soluble 3A102 VH bound to the FRα-expressing (A) MDA-MB-231, A549 and H292 cells and (B) ECL-08, -10, -12, -16, -17, and -20, NSCLC patient-derived primary cancer cells compared to the BJ cells. Data are shown as the mean ± SD (*n* = 3). *p < 0.05 compared to the negative control (BJ).

**Figure 8.**
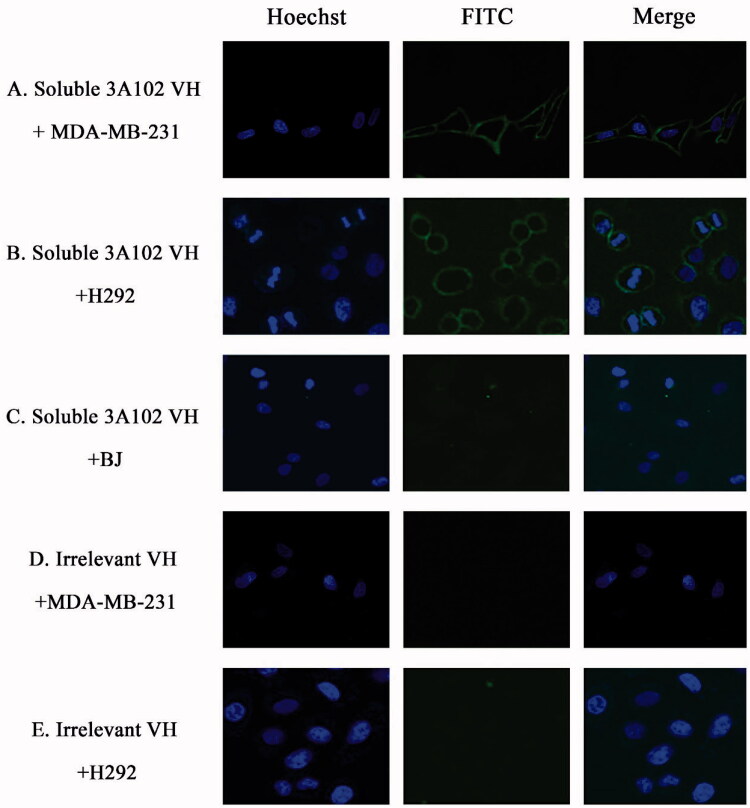
Representative cell images of soluble 3A102 VH binding, as determined by SLCM. (A, B and C) MDA-MB-231, H292 and BJ cells were stained with soluble 3A102 VH, respectively. (D and E) MDA-MB-231 and H292 cells were stained with an irrelevant VH, respectively. The antibody was detected with mouse anti-His-tag and goat anti-mouse IgG-FITC conjugate (green) Abs, respectively. Nuclei are labeled with Hoechst 33342 (blue).

MDA-MB-231, A549 and H292 were assessed for FRα expression by cell-based ELISA and cell IFA using a rabbit anti-hFRα polyclonal antibody. As shown in Supplemental Figure 1, 2, all cells expressed FRα on their surface, so they could be used to investigate the binding activity of soluble 3A102 VH against the nature form of FRα receptor. Then, we evaluated the binding ability of soluble 3A102 VH to FRα on the cell surface. Due to the soluble 3A102 VH Ab showed cross-reactivity with FRβ, the MDA-MB-231 cell that expresses only FRα but not FRβ isoform, was used to query the binding (Shen et al., 2018). After binding MDA-MB-231 with soluble 3A102 VH, a high intensity signal in cell-based ELISA was observed and also exhibited a fluorescence signal around the cell surface in IFA (Figure. 7(A) and 8(A)). These results supported the idea that the soluble 3A102 VH could bind to native conformation of FRα form on cell surface. Next, cell-based ELISA also was used to test the activity of soluble 3A102 VH toward the FRα on the NSCLC cell lines. A significant and dose-dependent difference in the A_450_ between the FRα expressing NSCLC cell line (A549 and H292) and non-FRα expressing (BJ) cells was evident after incubating with soluble 3A102 VH (Figure 7(A)). Moreover, after binding with soluble 3A102 VH, a high intensity signal (A_450_) was observed in the NSCLC patient-derived primary cancer cells compared to the BJ cells (Figure 7(B)). These results confirmed that the soluble 3A102 VH could bind with the native form of FRα on both NSCLC cells and NSCLC patient-derived primary cancer cells.

A cell IFA was also performed to confirm the targeting activity of the soluble 3A102 VH against the native form of FRα expressed on the cell surface of NSCLC cells. The H292 cells displayed a high fluorescence signal around the cell surface after being incubated with soluble 3A102 VH, while BJ displayed no obvious fluorescence signal (Figure 8(B,C)). Additionally, the irrelevant VH incubated with H292 cells revealed no fluorescence signal around the cells (Figure 8(E)). Both the cell ELISA and immunofluorescence results demonstrated that the soluble 3A102 VH retained its epitope binding specificity and targeting ability toward the native form of FRα.

### Cell internalization of soluble 3A102 VH

**Figure 9.**
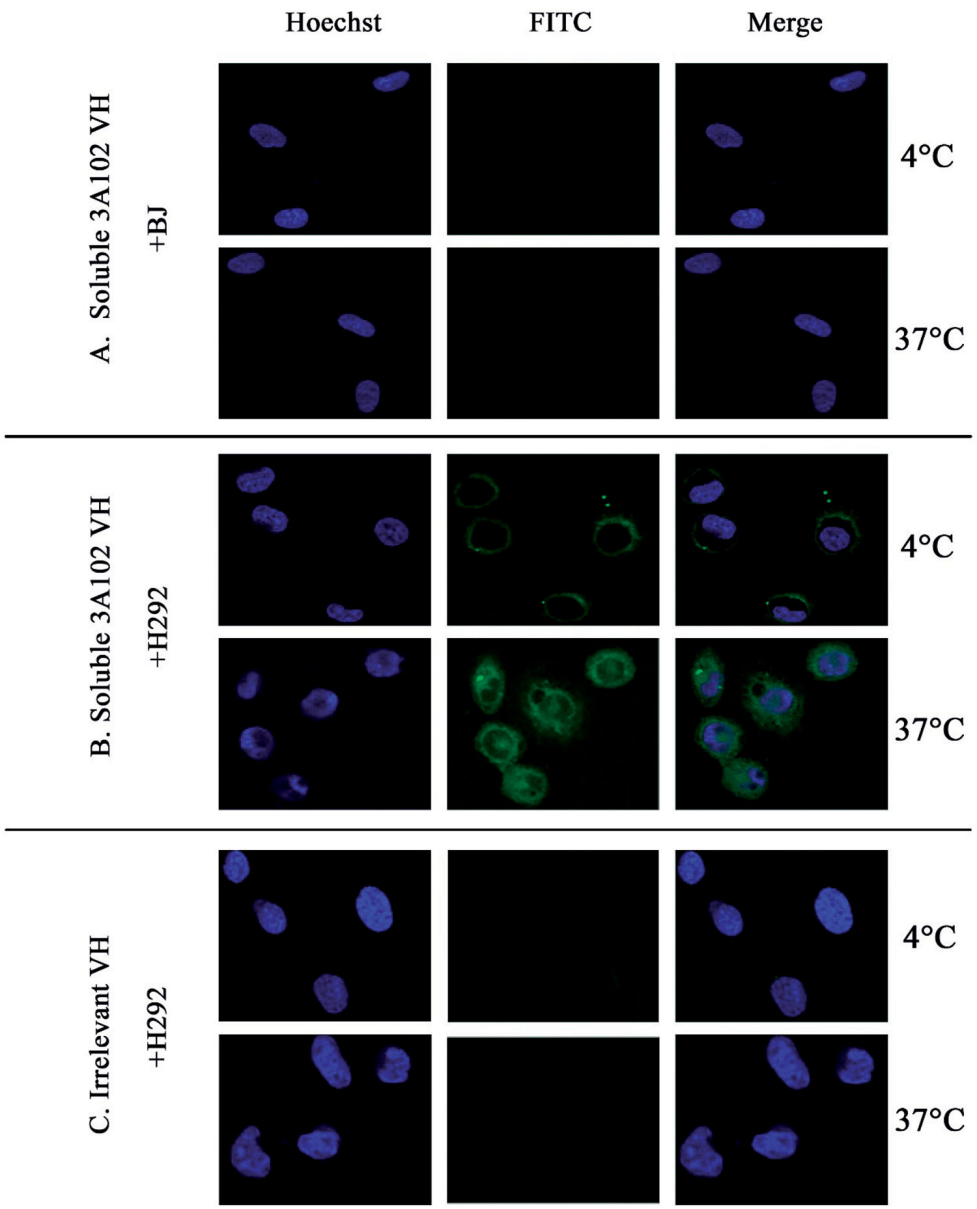
Cell internalization of soluble 3A102 VH as determined by SLCM. Representative images of (A and B) BJ and H292 cells stained with soluble 3A102 VH at 4 °C and 37 °C, respectively, and (C) H292 cells stained with the irrelevant VH at 4 °C and 37 °C. The antibody was detected with protein-A-FITC conjugate (green). Nuclei are labeled with Hoechst 33342 (blue).

To develop a suitable ADC treatment, the selected antibodies must have the ability to bind to and then induce internalization of the ADC into the cell for intracellular release of the cytotoxic drug. Thus, the soluble 3A102 VH was evaluated for its ability to be internalized into FRα-positive cells. Soluble 3A102 VH was incubated with BJ and H292 cells for 3 h at 37 °C, fixed, and then examined under SLCM to visualize the level of internalization of VH. At 4 °C in Figure 9(B), the internalization of soluble 3A102 VH was inhibited under the cold condition, resulting in signal fluorescence was observed only around the cell surface. While curing at 37 °C that introduced cell internalization, the fluorescence signal was observed in both cytoplasm and at perinuclear region of H292 cells (Figure 9(B)). These results revealed that soluble 3A102 VH could be internalized into H292 cells under inducing temperature. In contrast, neither BJ cells incubated with soluble 3A102 VH nor H292 cells incubated with the irrelevant VH showed no any binding or internalization into the cells both at 4 and 37 °C (Figure 9(A,C)).

### Discussion

Nowadays, targeted therapy is the preferred choice for the disease management of patients with NSCLC (Yuan et al., 2019). An ADC is one of the targeted treatment approaches that capitalizes on the highly specific targeting of mAbs to transport a drug into cancer cells, while minimizing the exposure to non-target tissues. However, the limitations of an ADC may be the incomplete penetration into cancer cells and solid tumor, due to the large size of the intact Ab (Xenaki et al., 2017). More recently, VH Abs from many species have emerged as a smaller targeting molecule (Kontermann et al., 2009; Hairul Bahara et al., 2016; Keyaerts et al. 2016; Teng et al., 2020). The idea behind these studies was based upon the advantages of VH of its small size resulting in a good penetration into the desired cells (Arbabi Ghahroudi et al., 1997; Harmsen & De Haard, 2007) . In addition, their lower immunogenicity than animal-derived VH, due to the high degree of identity of their framework to human Abs, makes human VH approaches preferred over xenogeneic Abs and encourages ADC development.

Another important molecule for targeted therapy is cancer-specific cell markers. In NSCLC, FRα has been a potential marker for both folic acid and FRα-specific Abs to develop targeted therapy. However, folic acid-drug conjugates have the major concern that they can be transported into normal cells via other pathways beside the FR, such as the folate carrier and the proton-coupled folate transporter, which results in the uptake of the payload drug by normal cells (Wibowo et al., 2013; Goldman et al., 2010). For this reason, we aimed to develop a novel human VH Ab against the FRα and established its targeting ability in preliminary *in vitro* testing in this study. We applied bio-panning to select for human synthetic VH Abs from a Dab phage library synthesized by PCR mutagenesis of amino acids in the CDR1-3 region to generate a variety of VH Ab repertoires (Lee et al., 2007).

To screen the VH Abs against FRα, we used a rhFRα protein as the ELISA coating antigen, since it’s a simple and widely accepted method for successful selection of antigen-specific Abs (Barkhordarian et al., 2006; Lim et al., 2019). To select specific phages, we increased the stringency in each of the seven rounds of the bio-panning process. First, we used a high concentration of rhFRα as the coated antigen in the first round of bio-panning to prevent the loss of specific phages if the antigen had been deformed after being coated on the solid surface or was removed during washing. Then, in subsequent rounds, the amount of antigen (rhFRα) was decreased to keep only phage clones with a high specificity to rhFRα, while the number of washes and the concentration of Tween-20 in the wash buffer was increased in each round of bio-panning to eliminate nonspecific phages.

At the end of the bio-panning, we selected the positive 3A102 VH phage clone as it showed the highest binding ability to rhFRα protein. In fact, FRs consist of three subtypes: FRα and FRβ, which are extracellular receptors anchored to the membranes (Wibowo et al., 2013), and the gramma folate receptor (FRγ), which is a soluble receptor and secreted at low levels from lymphoid cells in the spleen, thymus, and bone marrow (Wibowo et al., 2013). Accordingly, FRγ is unsuitable as a TAA surface maker and so we only tested the cross-reactivity between rhFRα and rhFRβ. Interestingly, the 3A102 VH Ab also showed cross-reactivity with FRβ. This result was consistent with previous research where a hybridoma derived mAb that recognized FRα also bound to FRβ (Nagai et al., 2015). Cross-reactivity between these two antigens occurred because FRβ has a high sequence similarity (77%) with FRα (Wibowo et al., 2013). Since FRβ is only highly expressed in leukemia, lymphomas, and the tumor-associated macrophages (TAM) in NSCLC, liver, breast, and brain cancers (Shen et al., 2015), these facts highlight the potential of 3A102 VH for targeting against the TAMs of NSCLC and other cancers as well.

Nucleotide sequencing identified that 3A102 VH had a translational defect codon in the CDRs, namely amber stop codons (UAG), frequently found in phage displayed Abs due to the randomization of CDR sequences. This codon stops protein expression in a non-amber suppressor *E. coli.* strain, but could be read as glutamine (CAG) instead of a stop codon in an amber suppressor *E. coli* strain, such as TG1 (Carmen & Jermutus, 2002). Consequently, the amber codon of 3A102 VH was optimized in CDR3 to encode for glutamine before reforming from phage to soluble protein in *E. coli*. Since the VH Ab has one pair of disulfide bonds that play a significant role in the protein folding and stability, the VH was then selected to be expressed as soluble protein in *E. coli* Shuffle, an engineered strain that can promote disulfide bond formation in its cytoplasmic part (Liu & May, 2012; Lobstein et al., 2012).

After expression and purification, the soluble 3A102 VH still retained its bioactivity against FRα. Cell-based ELISA was performed to evaluate the activity of soluble 3A102 VH to the native conformation of FRα on NSCLC cell lines and NSCLC patient-derived primary cancer cells. With a strong signal against FRα, this suggested that soluble 3A102 VH could bind to the native form of FRα on both cell lines. The K_aff_ value of the soluble 3A102 VH against FRα was around 7.77 ± 0.25 × 10^7^ M ^− 1^, corresponding to several previous reports that have described VH Abs with affinities achieved in the range from 10^7^–10^10^ M ^− 1^, as shown in Supplemental Table 1. However, the K_aff_ value of soluble 3A102 VH described here was rather lower than that of intact monoclonal antibodies, having a K_aff_ value higher than 10^7^ M ^− 1^ (Ohlin et al., 1991; Michael et al., 1998; Bayat et al., 2013; Lee et al., 2017). To improve the affinity of soluble 3A102 VH, site-directed mutagenesis or the generation of dimer formed through VH-VH non-covalent interactions were taken into consideration (Adams et al., 1998; Baral et al., 2012; Li et al., 2014). The key features to develop an ADC is that the VH should have targeting and internalization abilities into target cells, so as to release the cytotoxic payload drug inside the tumor. So, these two key abilities were intentionally determined in our study.

Cell IFAs revealed that the 3A102 VH retained its targeting ability around the cell surface of NSCLC cells, and that it could bind to and become internalized into FRα-expressing cell lines. A previous study reported that only high affinity Abs could exhibit a high degree of internalization in both *in vitro* and *in vivo* studies (Rudnick et al., 2011). Therefore, the observed binding affinity of about 7.77 ± 0.25 × 10^7^ M ^− 1^ could be high enough to promote the internalization of VH into the FRα-expressing cells.

Recently, Bannas et al. demonstrated a good targeting property of 16aVHH, a llama antibody directed against ARTC2 in lymphoma cells. Their small VH fragment showed a faster and deeper tumor penetration and a higher tumor to background ratio than intact antibody (Bannas et al., 2015). These evidences may imply the potential of VH antibody fragment in order to be a promising targeting molecule and may improve ADC efficacy of current FRα specific intact antibodies.

Currently, there has been a rapid growth in the number of anti-cancer agents, such as Renieramycin M from the Thai blue sponge *Xestospongia sp.* that was reported for its anti-cancer activity against both normal cells and NSCLC cell lines (Sirimangkalakitti et al., 2016). To improve the specific therapeutic ability of these new findings, our soluble 3A102 VH could be offered. In conclusion, this our preliminary *in vitro* study provided a good rationale for using a phage library to isolate a novel human VH as a targeting molecule against FRα. However, the targeting ability and *in vivo* stability of soluble 3A102 VH, including the drug-conjugate toxicity, should be suggested and warrants further investigation

## References

[CIT0001] Ab O, Whiteman KR, Bartle LM, et al. (2015). IMGN853, a Folate Receptor-α (FRα)-Targeting Antibody-Drug Conjugate, Exhibits Potent Targeted Antitumor Activity against FRα-Expressing Tumors. Mol Cancer Ther 14:1605–13. 25904506 10.1158/1535-7163.MCT-14-1095

[CIT0002] Adams GP, Schier R, Marshall K, et al. (1998). Increased affinity leads to improved selective tumor delivery of single-chain Fv antibodies. Cancer Res 58:485–90. 9458094

[CIT0003] Ahmad I, Iwata T, Leung HY. (2012). Mechanisms of FGFR-mediated carcinogenesis. Biochim Biophys Acta 1823:850–60. 22273505 10.1016/j.bbamcr.2012.01.004

[CIT0004] Allard J, Risinger J, Morrison C, et al. (2007). Overexpression of folate binding protein is associated with shortened progression-free survival in uterine adenocarcinomas. Gynecol Oncol 107:52–7. 17582475 10.1016/j.ygyno.2007.05.018

[CIT0005] Arbabi Ghahroudi M, Desmyter A, Wyns L, et al. (1997). Selection and identification of single domain antibody fragments from camel heavy-chain antibodies. FEBS Lett 414:521–6. 9323027 10.1016/s0014-5793(97)01062-4

[CIT0006] Azzazy HM, Highsmith WE. Jr. (2002). Phage display technology: clinical applications and recent innovations. Clin Biochem 35:425–45. 12413604 10.1016/s0009-9120(02)00343-0

[CIT0007] Bannas P, Lenz A, Kunick V, et al. (2015). Molecular imaging of tumors with nanobodies and antibodies: Timing and dosage are crucial factors for improved in vivo detection. Contrast Media Mol Imaging 10:367–78. 25925493 10.1002/cmmi.1637

[CIT0008] Baral TN, Chao SY, Li S, et al. (2012). Crystal structure of a human single domain antibody dimer formed through V(H)-V(H) non-covalent interactions. PLoS One 7:e30149. 22253912 10.1371/journal.pone.0030149 PMC3257273

[CIT0009] Barkhordarian H, Emadi S, Schulz P, et al. (2006). Isolating recombinant antibodies against specific protein morphologies using atomic force microscopy and phage display technologies. Protein Eng Design Select PEDS 19:497–502. 10.1093/protein/gzl036 16984950

[CIT0010] Bates A, David PC. vs. Goliath: (2019 Apr 9). The structure, function, and clinical prospects of antibody fragments. Antibodies. 8:28. 10.3390/antib8020028 PMC6640713 31544834

[CIT0011] Bayat AA, Yeganeh O, Ghods R, et al. (2013). Production and characterization of a murine monoclonal antibody against human ferritin. Avicenna J Med Biotechnol 5:212–219. 24285995 PMC3838765

[CIT0012] Bazan J, Całkosiński I, Gamian A. (2012). Phage display–a powerful technique for immunotherapy: 1. Introduction and Potential of Therapeutic Applications. Human Vaccines & Immunotherapeutics 8:1817–28. 22906939 10.4161/hv.21703 PMC3656071

[CIT0013] Beatty JD, Beatty BG, Vlahos WG. (1987). Measurement of monoclonal antibody affinity by non-competitive enzyme immunoassay. J Immunol Methods 100:173–9. 2439600 10.1016/0022-1759(87)90187-6

[CIT0014] Brown Jones M, Neuper C, Clayton A, et al. (2008). Rationale for folate receptor alpha targeted therapy in "high risk" endometrial carcinomas. Int J Cancer 123:1699–703. 18646191 10.1002/ijc.23686

[CIT0015] Carmen S, Jermutus L. (2002). Concepts in antibody phage display. Brief Funct Genomic Proteomic 1:189–203. 15239904 10.1093/bfgp/1.2.189

[CIT0016] Davies J, Riechmann L. (1996). Single antibody domains as small recognition units: design and in vitro antigen selection of camelized, human VH domains with improved protein stability. Protein Eng 9:531–7. 8862554 10.1093/protein/9.6.531

[CIT0017] Fernández M, Javaid F, Chudasama V. (2018). Advances in targeting the folate receptor in the treatment/imaging of cancers. Chem Sci 9:790–810. 29675145 10.1039/c7sc04004k PMC5890329

[CIT0018] Furler RL, Nixon DF, Brantner CA, et al. (2018). TGF-β Sustains Tumor Progression through Biochemical and Mechanical Signal Transduction. Cancers 10:199. 10.3390/cancers10060199 PMC6025279 29903994

[CIT0019] Goldman ID, Chattopadhyay S, Zhao R, et al. (2010). The antifolates: evolution, new agents in the clinic, and how targeting delivery via specific membrane transporters is driving the development of a next generation of folate analogs. Curr Opin Investigational Drugs. 11:1409–23. 21154123

[CIT0020] Hairul Bahara NH, Chin ST, Choong YS, et al. (2016). Construction of a semisynthetic human VH single-domain antibody library and selection of domain antibodies against α-crystalline of mycobacterium tuberculosis. J Biomol Screen 21:35–43. 26423338 10.1177/1087057115609144

[CIT0021] Harmsen MM, De Haard HJ. (2007). Properties, production, and applications of camelid single-domain antibody fragments. Appl Microbiol Biotechnol 77:13–22. 17704915 10.1007/s00253-007-1142-2 PMC2039825

[CIT0022] Hartmann LC, Keeney GL, Lingle WL, et al. (2007). Folate receptor overexpression is associated with poor outcome in breast cancer. Int J Cancer 121:938–42. 17487842 10.1002/ijc.22811

[CIT0023] Iqbal N, Iqbal N. (2014). Human Epidermal Growth Factor Receptor 2 (HER2) in cancers: overexpression and therapeutic implications. Mol Biol Inter. 2014:852748. 10.1155/2014/852748 PMC4170925 25276427

[CIT0024] Iwakiri S, Sonobe M, Nagai S, et al. (2008). Expression status of folate receptor alpha is significantly correlated with prognosis in non-small-cell lung cancers. Ann Surg Oncol 15:889–99. 18181001 10.1245/s10434-007-9755-3

[CIT0025] Jäger D, Jäger E, Knuth A. (2001). Immune responses to tumour antigens: implications for antigen specific immunotherapy of cancer. J Clin Pathol 54:669–74. 11533070 10.1136/jcp.54.9.669 PMC1731514

[CIT0026] Kalli KR, Oberg AL, Keeney GL, et al. (2008). Folate receptor alpha as a tumor target in epithelial ovarian cancer. Gynecol Oncol 108:619–26. 18222534 10.1016/j.ygyno.2007.11.020 PMC2707764

[CIT0027] Keyaerts M, Xavier C, Heemskerk J, et al. (2016). Phase I study of 68Ga-HER2-nanobody for PET/CT assessment of HER2 expression in breast carcinoma. J Nucl Med 57:27–33. 26449837 10.2967/jnumed.115.162024

[CIT0028] Konner JA, Bell-McGuinn KM, Sabbatini P, et al. (2010). Farletuzumab, a humanized monoclonal antibody against folate receptor alpha, in epithelial ovarian cancer: a phase I study. Clin Cancer Res 16:5288–95. 20855460 10.1158/1078-0432.CCR-10-0700

[CIT0029] Kontermann RE, Scheurich P, Pfizenmaier K. (2009). Antagonists of TNF action: clinical experience and new developments. Expert Opin Drug Discov 4:279–92. 23489126 10.1517/17460440902785167

[CIT0030] Kovtun YV, Audette CA, Ye Y, et al. (2006). Antibody-drug conjugates designed to eradicate tumors with homogeneous and heterogeneous expression of the target antigen. Cancer Res 66:3214–21. 16540673 10.1158/0008-5472.CAN-05-3973

[CIT0031] Lambert JM. (2013). Drug-conjugated antibodies for the treatment of cancer. Br J Clin Pharmacol 76:248–62. 23173552 10.1111/bcp.12044 PMC3731599

[CIT0032] Lee CM, Iorno N, Sierro F, et al. (2007). Selection of human antibody fragments by phage display. Nat Protoc 2:3001–8. 18007636 10.1038/nprot.2007.448

[CIT0033] Lee H-J, Lee J-Y, Park M-H, et al. (2017). Monoclonal antibody against G Glycoprotein increases respiratory syncytial virus clearance in vivo and prevents vaccine-enhanced diseases. PLoS One 12:e0169139–e0169139. 28076422 10.1371/journal.pone.0169139 PMC5226777

[CIT0034] Li B, Fouts AE, Stengel K, et al. (2014). In vitro affinity maturation of a natural human antibody overcomes a barrier to in vivo affinity maturation. MAbs 6:437–45. 24492299 10.4161/mabs.27875 PMC3984332

[CIT0035] Lim CC, Woo PCY, Lim TS. (2019). Development of a Phage display panning strategy utilizing crude antigens: isolation of MERS-CoV nucleoprotein human antibodies. Sci Rep 9:6088. 30988390 10.1038/s41598-019-42628-6 PMC6465254

[CIT0036] Liu H, May K. (2012). Disulfide bond structures of IgG molecules: structural variations, chemical modifications and possible impacts to stability and biological function. MAbs 4:17–23. 22327427 10.4161/mabs.4.1.18347 PMC3338938

[CIT0037] Lobstein J, Emrich CA, Jeans C, et al. (2012). SHuffle, a novel Escherichia coli protein expression strain capable of correctly folding disulfide bonded proteins in its cytoplasm. Microb Cell Fact 11:56. 22569138 10.1186/1475-2859-11-56 PMC3526497

[CIT0038] Michael N, Accavitti MA, Masteller E, et al. (1998). The antigen-binding characteristics of mAbs derived from in vivo priming of avian B cells. Proc Natl Acad Sci U S A 95:1166–1171. 9448303 10.1073/pnas.95.3.1166 PMC18708

[CIT0039] Moore KN, Martin LP, O'Malley DM, et al. (2018). A review of mirvetuximab soravtansine in the treatment of platinum-resistant ovarian cancer. Future Oncology (London, England 14:123–36. 10.2217/fon-2017-0379 29098867

[CIT0040] Nagai T, Furusho Y, Li H, et al. (2015). Production of a high-affinity monoclonal antibody reactive with folate receptors alpha and beta. Monoclon Antib Immunodiagn Immunother 34:181–90. 26090596 10.1089/mab.2014.0072

[CIT0041] Ohlin M, Sundqvist VA, Gilljam G, et al. (1991). Characterization of human monoclonal antibodies directed against the pp65-kD matrix antigen of human cytomegalovirus. Clin Exp Immunol 84:508–514. 1710548 PMC1535436

[CIT0042] Patel NR, Piroyan A, Nack AH, et al. (2016). Design, synthesis, and characterization of folate-targeted platinum-loaded theranostic nanoemulsions for therapy and imaging of ovarian cancer. Mol Pharm 13:1996–2009. 27170232 10.1021/acs.molpharmaceut.6b00149

[CIT0043] Ponte JF, Ab O, Lanieri L, et al. (2016). Mirvetuximab Soravtansine (IMGN853), a Folate Receptor Alpha-Targeting Antibody-Drug Conjugate, Potentiates the Activity of Standard of Care Therapeutics in Ovarian Cancer Models. Neoplasia (New York, NY) 18:775–84. 10.1016/j.neo.2016.11.002 PMC5126132 27889646

[CIT0044] Rodriguez-Fernandez S, Murillo M, Villalba A, et al. (2019). Impaired Phagocytosis in Dendritic Cells From Pediatric Patients With Type 1 Diabetes Does Not Hamper Their Tolerogenic Potential. Front. Immunol 10:2811. 31849983 10.3389/fimmu.2019.02811 PMC6892968

[CIT0045] Rudnick SI, Lou J, Shaller CC, et al. (2011). Influence of affinity and antigen internalization on the uptake and penetration of Anti-HER2 antibodies in solid tumors. Cancer Res 71:2250–2259. 21406401 10.1158/0008-5472.CAN-10-2277 PMC3077882

[CIT0046] Sato S, Itamochi H. (2016). Profile of farletuzumab and its potential in the treatment of solid tumors. Onco Targets Ther 9:1181–8. 27022278 10.2147/OTT.S98242 PMC4789847

[CIT0047] Shen J, Hu Y, Putt KS, et al. (2018). Assessment of folate receptor alpha and beta expression in selection of lung and pancreatic cancer patients for receptor targeted therapies. Oncotarget 12:4485–95. 10.18632/oncotarget.23321 PMC5796989 29435118

[CIT0048] Shen J, Putt KS, Visscher DW, et al. (2015). Assessment of folate receptor-β expression in human neoplastic tissues. Oncotarget 6:14700–9. 25909292 10.18632/oncotarget.3739 PMC4546498

[CIT0049] Shi H, Liu L, Wang Z. (2013). Improving the efficacy and safety of engineered T cell therapy for cancer. Cancer Lett 328:191–7. 23022475 10.1016/j.canlet.2012.09.015

[CIT0050] Siegel RL, Miller KD, Jemal A. (2017). Cancer statistics, 2017. CA Cancer J Clin 67:7–30. 28055103 10.3322/caac.21387

[CIT0051] Sirimangkalakitti N, Chamni S, Charupant K, et al. (2016). Chemistry of Renieramycins. 15. Synthesis of 22-O-Ester Derivatives of Jorunnamycin A and Their Cytotoxicity against Non-Small-Cell Lung Cancer Cells. J Nat Prod 79:2089–93. 27487087 10.1021/acs.jnatprod.6b00433

[CIT0052] Sokolowska-Wedzina A, Chodaczek G, Chudzian J, et al. (2017). High-affinity internalizing human scFv-Fc antibody for targeting FGFR1-overexpressing lung cancer. Mol Cancer Res 15:1040–50. 28483948 10.1158/1541-7786.MCR-16-0136

[CIT0053] Srinivasarao M, Galliford CV, Low PS. (2015). Principles in the design of ligand-targeted cancer therapeutics and imaging agents. Nat Rev Drug Discov 14:203–19. 25698644 10.1038/nrd4519

[CIT0054] Syrkina MS, Vassetzky YS, Rubtsov MA. (2019). MUC1 Story: Great Expectations, Disappointments and the Renaissance. Curr Med Chem 26:554–63. 28820070 10.2174/0929867324666170817151954

[CIT0055] Tamura T, Kurishima K, Nakazawa K, et al. (2015). Specific organ metastases and survival in metastatic non-small-cell lung cancer. Mol Clin Oncol 3:217–21. 25469298 10.3892/mco.2014.410 PMC4251107

[CIT0056] Teng Y, Young JL, Edwards B, et al. (2020). Diverse human V(H) antibody fragments with bio-therapeutic properties from the Crescendo Mouse. New Biotechnol 55:65–76. 10.1016/j.nbt.2019.10.003 31600579

[CIT0057] Thundimadathil J. (2012). Cancer treatment using peptides: current therapies and future prospects. J Amino Acids 2012:967347. 23316341 10.1155/2012/967347 PMC3539351

[CIT0058] Toffoli G, Russo A, Gallo A, et al. (1998). Expression of folate binding protein as a prognostic factor for response to platinum-containing chemotherapy and survival in human ovarian cancer. Int J Cancer 79:121–6. 9583724 10.1002/(sici)1097-0215(19980417)79:2<121::aid-ijc4>3.0.co;2-v

[CIT0059] Wibowo AS, Singh M, Reeder KM, et al. Structures of human folate receptors reveal biological trafficking states and diversity in folate and antifolate recognition. Proceedings of the National Academy of Sciences of the United States of America. 2013;110(38):15180–8. 23934049 10.1073/pnas.1308827110 PMC3780903

[CIT0060] Woodman C, Vundu G, George A, et al. (2021). Applications and strategies in nanodiagnosis and nanotherapy in lung cancer. Semin Cancer Biol 69:349–64. 32088362 10.1016/j.semcancer.2020.02.009

[CIT0061] Xenaki KT, Oliveira S, van Bergen En Henegouwen PMP. (2017). Antibody or Antibody Fragments: Implications for Molecular Imaging and Targeted Therapy of Solid Tumors. Front. Immunol 8:1287. 29075266 10.3389/fimmu.2017.01287 PMC5643388

[CIT0062] Yuan M, Huang LL, Chen JH, et al. (2019). The emerging treatment landscape of targeted therapy in non-small-cell lung cancer. Signal Transduct Target Ther 4:61. 31871778 10.1038/s41392-019-0099-9 PMC6914774

